# Some Fallacies of the Bioplasson Theory

**Published:** 1882-10

**Authors:** Lester Curtis

**Affiliations:** Chicago


### Selections.

### SOME FALLACIES OF THE BIOPLASSON THEORY. A REPLY TO DR. HEITZMANN. By LESTER CURTIS, M. D., Chicago.

In the June number of the *Dental Cosmos* is an article written by Dr. C. Heitzmann, of New York, on "The Minute Anatomy of the Teeth in the Light of the Bioplasson Theory." It is chiefly an explanation of his peculiar views of cell-structure. The views may be summarized as follows: Every living organism contains in its interior "a delicate reticulum pervading the whole mass." This reticulum has no break anywhere, but is continuous throughout the whole body. It is the machine for the production of all motions, and is the only living part of the organism. There are no such things as cells. The appearances which have been called cells are only condensations of the net-work.

In the course of his article Dr. Heitzmann refers to a recent paper of mine. This paper was a review of his theory so far as relates to the red and white blood-corpuscles and the pus-corpuscle. I will recapitulate some of the chief points of the paper, in order that those who have not read it may the better appreciate Dr. Heitzmann's criticisms.

About three years ago, before I began to study the net-work, I wrote to Dr. Heitzmann and asked him the best method of seeing the structure and the kind and quality of glass necessary to show it. His recommendations were as follows:

"Take a drop of pus, fresh, without adding anything, and you will see the wonderful structure in each pus-corpuscle with great ease.

"Prick your skin on the palmar surface of your thumb, transport the drop on a slide, and cover right away with a thin covering-glasss, the edges of which have been oiled, so as to prevent evaporation of the fluid. In the perfectly fresh blood you will see the structure in each colorless corpuscle.

"Add to a drop of fresh blood a small drop of a forty per cent. solution of bichromate of potash. This will within one hour extract the hemoglobin, and you must succeed in seeing the reticular structure in each red blood-corpuscle."

The glasses recommended for showing this structure with such ease were "a first-class $\frac{1}{10}$ immersion—such as I use—of Vèrick's, Hartnack's, Grunow's, and Tolles's manufacture." The list of names, though containing good ones, is slightly miscellaneous, and nothing is said about the need of anything more than ordinary water immersion-glasses. Indeed, some of these makers had not then, and have not yet, so far as I know, made any of the most modern homogeneous immersion objectives. Nothing is said about the need of any special appliances for illumination. It is probable, therefore, that my microscope, with a Powell & Leland dry condenser, and especially my Powell & Lealand water immersion $\frac{1}{16}$, is fully equal to the task of seeing the structure. With these appliances I have done the most of my work on this subject, though I have also used some of the very finest homogeneous glasses, including Zeis's, Tolles's, Powell & Lealand's new formula $\frac{1}{8}$, and others.

Proceeding according to his directions, I added a forty per cent. solution of bichromate of potash to a drop of blood, and studied the red corpuscles. Many of them lost their color, indeed; but with this loss of color they underwent some other remarkable changes. They became reduced in diameter from one-third to one-half, and their surfaces presented so many wrinkles and distortions that they were almost unrecognizable as blood-corpuscles. I saw no net-work in them, but even if I had seen something that looked like a net-work, I should have placed little reliance upon it as an indication of actual structure, when seen among so many unnatural appearances. I therefore turned from the red to the white corpuscles, following in my study the directions laid down by Dr. Heitzmann.

It so happened that when I began to study the white corpuscle I had been using the objective, with which I wished to study the corpuscle, in the examination of an object protected by a very thick cover. In preparing the specimen of blood I used for a cover a film of mica. The cover-adjustment had been left as it was arranged for this thick cover, and was, of course, wrong for the mica. When I had found a white corpuscle with a lower power, I put on the high power and brought the corpuscle into view. I could not refrain from an exclamation of surprise. There stood the net-work before me as plain as could be, and for an instant I thought that Dr. Heitzmann was right; but the body was blurred at the edges, and the red corpuscles in the field had a fuzzy indistinct outline. It then occurred to me that I had not arranged the cover-adjustment. I began to turn the adjustment; as I turned, the field of view became clearer, but the net-work grew fainter. Finally, when the field came out bright and clear, and the outlines of the red corpuscles were well defined and sharp the net-work was gone. In place of it the corpuscle was covered with little rounded eminences, varying in size and in distance from one another.

These eminences were always seen when the cover-adjustment was right. I had in my possession for some time a very fine homogeneous immersion $\frac{1}{8}$ made by Gundlach. This glass had no cover-adjustment; it always showed the eminences, and never the net-work. The eminences appear to be minute granules, of which the corpuscle is composed. They are seen with great distinctness in the large granular white corpuscles that are so abundant in anæmic persons. Women after prolonged lactation are excellent subjects in which to study them. In some of these cases the nodules are so distinct as to strikingly remind one of the zoo-glœa masses of bacteria spores that one may see at any time in urine beginning to decompose. In some cases the granules are scarcely less distinct than in the zooglæa mass. I have often seen the vacuoles, which so frequently form in the corpuscles, come so near the edge as to leave only one row of granules between the vacuoles and the edge of the corpuscle. Sometimes this thin rim breaks and leaves the granules projecting in a point. I have even seen some of the granules become detached and float away from the corpuscle. In some instances the granules leave the body in great numbers, and a shadowy, ghost-like structure remains, with here and there a granule sticking to it.

By keeping the slide with the blood upon it for ten or twelve hours, taking care that the edge of the cover is well oiled, the corpuscles change in appearance. The granules may then be seen to have a swarming motion, resembling that of a collection of bees.

Pus corpuscles resemble the white blood-corpuscles very closely. The corpuscles from an ordinary abscess resemble the white blood-corpuscle that has been kept for some hours. In pus-corpuscles the swarming motion of the granules is often very distinct. I have watched one particular granule in one of these bodies, and seen it move more than half way across the corpuscle before escaping from view. Occasionally I have seen pus-corpuscles in part of which the swarming motion could be seen, while it was absent in the other portions.

These granules within the corpuscles are of a size large enough to be easily measured under a power of from twelve to fifteen hundred diameters. They are also of an appreciable height, as can be seen under a sufficiently high power by gently changing the focus with the fine adjustment, and also by changing the direction of the illuminating-pencil, as can readily be done with the achromatic condenser.

My paper was illustrated with drawings. Part of these were made by myself from nature; the others were careful copies of drawings illustrating net-work made by Dr. Heitzmann or his friends.

All my own drawings were compared with the objects themselves by persons familiar with microscopic observation and were pronounced reasonably good likenesses. Drawings like these were sent to Dr. Heitzmann for his opinion. He criticised them in a letter to me in the following words: "You draw everything in and out of focus." Who will dare determine in every case just what is in focus and what is out of focus, and then venture to decide what to leave out of the drawing and what to put in? If Dr. Heitzmann's words mean anything at all, they mean,

"You should draw not what you really see, but what you think you ought to see."

I hope he is better able to reconcile this method with old-fashioned-honesty than I am. I insist that the only proper and honest way to draw is to draw everything exactly as you see it, without any change at all, and that is what I have tried to do in my figures.

The first of the illustrations of the net-work was made by Dr. Klein of London. It is commended in Dr. Heitzmann's article, and was referred to by him in a letter to me in the following words: "He draws the net-work *even nicer than it really appears.*" This disposes of the drawing as a representation of what was actually seen, and shows that it was constructed on Dr. Heitzmann's plan. The other drawings were made by Dr. Heitzmann himself.

My paper also contained arguments the bearing of which Dr. Heitzmann's imperfect knowledge of English may have prevented his perceiving. He, however, should know the details of a controversy upon the interpretation of the appearances seen upon some delicate silicious shells, which are used as tests for the microscope. One of these, called *pleurosigma angulatum*, was formerly described and figured as being covered with a honey-comb of cup-shaped figures, whose sides, united to each other, formed ribs, which ran across the surface of the shell. This was explained as a beautiful plan of nature to economize material and combine strength with lightness. But after a while glasses were improved, and the accuracy of this description was doubted. Then it was proved that, instead of being covered with ridges, the shell was really composed of little beads joined together so as to form a plate. The beads result from the peculiar manner in which silex is deposited from certain solutions; similar beads can be formed artificially. The hexagons, then, were the imperfectly-seen interspaces between the beads.

It is now a recognized fact that a series of circles placed close together will give rise to an optical illusion, and produce the appearance of hexagons bounded by straight lines. If these circles are of equal size and at uniform distances the hexagons will be regular, but if they are of irregular size and at unequal dis tances, the hexagons will be more or less distorted. We can, of course, distinguish the true shape of the figures if they are large enough and are placed in a good light near the eye; then they will appear distinct and perfectly round. But if they are removed to a little distance, especially if the light is dim, the illusion is so complete as to be almost irresistible, even when we know the shape of the figures. Any one may try this experiment for himself by drawing a series of circles and filling in the outlines with black.

The similarity of these hexagons to Dr. Heitzmann's figures is very striking. It would be of interest to know his explanation of the resemblance.

Most of us who have tried to push microscopic investigation are painfully aware that there is a limit of visibility beyond which we cannot go. The most difficult natural object which is used as a test of the quality of the microscope is another silicious shell called *amphipleura pellucida*. This shell is marked with lines $\frac{1}{90000}$ of an inch apart. When it was shown that these lines had wavy edges, which gave them an appearance somewhat like that of a rope, one of the most difficult of feats was thought to have been accomplished. From this appearance and the analogy of other similar structures it was inferred that the lines were rows of beads. Beads, then, $\frac{1}{90000}$ of an inch in diameter, are almost beyond the border of microscopic visibility, and are only to be seen with the very best of modern homogeneous immersion glasses, used with other modern accessories. But very few have been able to see them, even with these appliances.

Now, to return to Dr. Heitzmann's drawings. They represent the net-work at rest, actively dilated, and actively contracted In the one representing the corpuscle at rest the meshes of the net-work inclose spaces which average rather more than half an inch across. The white corpuscle in Dr. Klein's drawing is magnified not far from two thousand diameters. The meshes in Dr. Heitzmann's drawings are not less than five or ten times as large as in Dr. Klein's. If we may judge from these facts, then, Dr. Heitzmann's figures represent objects magnified *ten or twenty thousand diameters!* Let us look at these figures a little.

The nodal points at the intersection of the lines are largest in the drawing which represents the net-work as contracted. In this drawing the nodes are about one-fourth of an inch across. If we suppose the figure to be magnified twenty thousand diameters, the real size of the bodies would be $\frac{1}{80000}$ of an inch. But, not to press matters, say it is magnified only ten thousand diameters. This would give the bodies a size of $\frac{1}{40000}$ of an inch. Such a body might be seen without excessive difficulty with a good high-power glass. There are, however, some other difficulties that we may consider.

An ordinary white blood-corpuscle rarely, if ever, exceeds $\frac{1}{2000}$ of an inch in diameter. If these figures represent portions of such a corpuscle, then twenty of the nodules placed in a row would entend across the corpuscle, not counting the interspaces. But the interspaces, even in this figure, are about as large as the nodes. Turning to Dr. Klein's drawing, we see that between twenty and thirty nodes are found in the diameter of the body. Twenty nodes of $\frac{1}{40000}$ of an inch and twenty interspaces of the same size would be somewhat crowded in a space of $\frac{1}{2000}$ of an inch.

In the drawing showing the net-work in a relaxed condition, the nodal points are only one-sixteenth of an inch across. Here we are in a much better condition as regards room, but there arises another serious difficulty. If we still suppose that our corpuscle is magnified only ten thousand times, we shall have a $\frac{1}{160000}$ of an inch across, about one and three-fourth times smaller than those beads of *amphipleura pellucida*, which are so small as never to have been clearly seen.

Again, the widest of the lines which connect these nodes are less than the thirty-second of an inch across, and the narrowest much less than this. Does he really expect us to believe that he, or any one else, can see lines less than $\frac{1}{320000}$ of an inch across, and lines, too, exceedingly pale, and buried in a mass of tissue like them in color and appearance?

I might continue this subject further, and call attention to some investigations of Professor Abbe, of Jena, on the ultimate visibility of objects, which would afford food for reflection in this connection; but I will leave the point.

Such are some of my observations and reflections on this subject. While engaged in the study I have communicated, personally or by letter, with a large number of microscopists, several of whom have earned more than a national reputation. Some were kind enough to go over the ground together with me and by themselves. Every one of them, without exception, who did so agrees with me. Some have expressed themselves quite strongly on the point, and I have never succeeded in finding an experienced microscopist who believes the theory.

My paper was read at the last meeting of the American Society of Microscopists. There were present a number of lifelong microscopists, some of them men of eminence. The paper was received by them with marked favor, and without one dissenting voice, so far as I know.

In spite of this support which the subject has received, the following is all that Dr. Heitzmann has to say about it:

"Lately Dr. Lester Curtis published an article on this subject, and stated that he could not see the reticulum. This is a very modest way to announce that one cannot see what another can. If a person publicly confesses that he cannot play on the piano a master piece of Liszt's, we are willing to believe it; but does it follow that others cannot play the piece either? What is gained by such a confession? Dr. Curtis gives illustrations of what Elsberg and I have said and seen, and of what he could *not* see. I mention this because you are all prepared to understand that delicate observations of this nature are not easily made by every one. A great many look into the microscope, but very few can *see*. If one of you who had never played the violin were handed a good violin and told to play a tune, he would say, 'I can't.' Let him practice for a few years, and learn to play a tune. It is very much the same with the microscope. If you look in for but a short time, you cannot see. It required more than fifteen years' application to enable me to see what can readily be seen, and if a tyro comes and declares that he cannot see, I do not think that such assertions should be taken as proofs against facts corroborated by others."

I submit to the judgment of all fair-minded readers whether such a rejoinder is in any sense an answer to my arguments.

And I insist that until they are answered I have sufficient ground for holding that this net-work is merely the misinterpreted interspace between the granules, and that it is one more example of the many optical illusions which, from before Ehrenberg's day up to the present time, have so often led astray even good observers.

The plate herewith accompanied the paper referred to in the text.

Figs. 1 to 5 were drawn by myself.

### EXPLANATION OF THE PLATE.

Fig. 1. An ordinary white blood-corpuscle with one red corpuscle.

Fig. 2. An ordinary white blood-corpuscle from another person, and two red corpuscles lying one over the other.

Fig. 3. Granular white corpuscle from Mr. Griscom; granules beginning to leave the body.

Figs. 4 and 5. Granular corpuscles from Mr. Griscom, discharging their granules.

Fig. 6. A white blood-corpuscle, referred to by Dr. Heitzmann; from Klein's "Atlas of Histology."

Figs. 7, 8 and 9. Representation of the net-work from "The Structure and other Characteristics of Colored Blood-Corpuscles," by Louis Elsberg, page 46; referred to by Dr. Heitzmann. Fig. 7 represents the net-work at rest; Fig. 8, in extreme contraction; Fig. 9, in extreme extension.

